# Recent advances and challenges in antibacterial drug development

**DOI:** 10.5599/admet.1271

**Published:** 2022-03-04

**Authors:** Valeria Gigante, Hatim Sati, Peter Beyer

**Affiliations:** Impact Initiatives and Research Coordination Unit, Global Coordination Department, AMR Division, WHO, Geneva, Switzerland

**Keywords:** Antimicrobial Resistance, WHO Pipeline, Research and Development, Public Health

Antimicrobial resistance (AMR) is one of the leading causes of death globally. Recent estimates reveal that 1.2 million deaths were due to resistant bacterial infections in 2019 and 4.95 million deaths were associated with resistant bacterial infections [1]. There are some evidences suggesting that the current global crisis due to COVID-19 pandemic might have exacerbated antibiotic use. In addition, the pandemic has marginalized many public health priorities and programs in countries, resulting in services and programs interruption such as disease surveillance. This all together threatens the gains made in recent years against AMR. This includes nourishing the pipeline, investing in the development of new therapies knowing that research and development (R&D) of safe and effective products takes time, and adopting policy at the government level such as advanced remuneration frameworks to create a viable environment for new products [2].

Since 2017 World Health Organization (WHO) analyses the antibacterial development pipeline annually. The analysis covers traditional direct-acting small molecules and nontraditional antibacterial agents in clinical and preclinical development worldwide. The aim is to evaluate to what extent does the present pipeline address the WHO bacterial priority pathogens, *Mycobacterium tuberculosis* and *Clostridium difficile* [3]. The end goal is to understand the state of the pipeline and to direct R&D towards public health unmet needs. Today WHO pipeline review is the only global analysis that regularly covers antibacterial agents in development by large pharmaceutical companies as well as by medium and small size enterprises [4]. Findings from this analysis are featured in an annual WHO publication and the data are also made public through WHO R&D Observatory [5] as well as the Global AMR R&D Hub. Overall, this information allows for secondary analyses such as the estimation of the source and number of investments across the different development phases as shown in the Global AMR R&D Hub dashboard [6,7].

## The long road to approval

Between July 2017 and November 2021, twelve new antibacterial agents have been approved by either the US Food and Drug Administration (FDA), the European Medicines Agency (EMA), or both. Only one of these was approved in the last two years. This translates to an average duration of approximately 10.1 years for a new antimicrobial agent to advance from the discovery and preclinical stage until the market authorization [8]. Looking at the progression rate, a WHO Financial Model estimates that an antibacterial product in preclinical development has approximately a 12.5 % chance of successfully moving past the registration phase [8]. These estimations reflect the scientific complexity of developing new drug. Several factors make the development of innovative antibacterials specifically challenging. These include the limited funding, and the limited return on investment in the current volume-driven revenue models. This is exacerbated by the current epidemic that has slowed down and even halted many clinical and preclinical programmes.

However, COVID-19 could be looked at as an opportunity to push for ad-hoc regulatory pathways, sustainable public financial models, and other financial incentives that prioritize antibacterial agents as a finite public resource. This push could also include investments in new emerging technologies and nontraditional approaches such as synthetic mRNAs/antimicrobial peptides (AMPs) and phage therapies.

## Surviving the market and proving the value

Evaluating how the newly approved products address global public health priorities, only one agent, cefiderocol, is said to be active against all three “critical” Gram-negative bacteria. Almost 50 % of the newly approved agents (n = 5) target one priority pathogen in the critical category, carbapenem-resistant *Enterobacterales* (CRE). This leaves two critical targets; carbapenem-resistant *Acinetobacter baumannii* (CRAB) and carbapenem-resistant *Pseudomonas aeruginosa* (CRPA), largely unaddressed at present.

With some exceptions, all newly approved agents present limited clinical benefit over existing treatment. Over 80 % of the newly approved antibacterials belong to existing classes (mostly ß- lactamases) where resistance mechanisms are well established, and the development of resistance is expected.

The evident challenge to design new antibacterial classes calls for prudent use of the existing agents to ensure their longevity. In fact, the majority (7 out of 12) of the newly approved antibiotics are classified as “Reserve” according to the most recent WHO AWaRe classification, while three are in the “Watch” group [9,10]. One agent, contezolid has not been classified at the time of this publication, but like other oxazolidinones, is expected to be included in the “reserve” group in the next iteration of the AWaRe classification [10]. This also indicates that most new agents presently in development, when approved, will likely be in the reserve group of antibiotics to be used only when other treatment lines have failed.

This need to preserve new agents is another challenge to antibacterial development that could further limit investments. It also highlights the need for universal decoupled public financial model(s) through existing push or new pull mechanisms for antibacterial drug development. However, to justify public funding, antimicrobial programs need to demonstrate potential clinical utility of candidates and ideally novelty, by moving away from the development of worn derivatives with limited potential for clinical differentiation.

## Innovation in traditional antibacterial agents

Traditional antibacterial agents are defined as small-molecule agents that directly target bacteria to either halt their growth (bacteriostatic effect) or to kill them (bactericidal effect) [11]. The current clinical antibacterial pipeline counts approximately 78 antibacterial agents containing either new therapeutic entity or combinations of existing molecules with at least one new therapeutic entity. Among the 78 products, 45 are traditional antibacterial agents and 33 are nontraditional agents. Of the 45 traditional antibiotics, 27 (60 %) are reported to be active against the WHO bacterial priority pathogens, 13 (31.1 %) against *Mycobacterium tuberculosis* (TB) and five (11.1 %) against *Clostridium difficile* [5].

Innovation is a key aspect evaluated when reviewing antibacterial agents in clinical development. There is a lack of consensus on what qualifies as innovative antibacterial agent. WHO looks for evidence of innovation in: the proposed class; the mechanism of action; the molecular target; and the absence of cross-resistance. [5].

Considering the chemical class and the molecular target, among the 27 agents identified against the WHO critical priority pathogens, most of the antimicrobial agents in the clinical pipeline are derivatives of existing classes and β-lactams-β-lactamase inhibitors (BLIs) accounts for over 40 % (12/27) of these agents. Most of these combinations are active against Class A, and C and some are active against class D enzymes. Very few are reported to target Class B enzymes (MBLs). Both *Pseudomonas aeruginosa* and, to a certain extent, *Acinetobacter baumannii*, have developed resistance mechanisms beyond the production of β-lactamases, including decreased permeability of the outer membrane and upregulation of efflux pumps and modified penicillin-binding protein (PBP). This may explain the copious β-lactams-BLIs combinations approach found in the pipeline, which attempts to overcome these resistance mechanisms challenges.

However, this approach also reflects a lack of innovation in terms of new chemical classes and may result in many “me too” agents with limited potential for clinical differentiation. In fact, examining all the 27 traditional agents under development against WHO bacterial priority pathogens, only a few compounds in clinical development satisfy at least one of the innovation criteria described and address at the same time critical Gram-negative bacteria (CRAB, CRPA or CRE).

The anti-TB clinical antibacterial pipeline, on the other hand, looks more innovative, with seven (53 %) agents showing new chemical structures.

It is important to point out here that for any given agent in development, meeting one or more of the innovation criteria described must be interpreted in the context of clinical and public health unmet needs.

## Nontraditional agents, new approaches, same developmental pathway

“Nontraditional antimicrobials” refers to therapies that are not small molecules and/or aim at a nontraditional target and include a broad range of agents [12]. Since 2019, the WHO antibacterial pipeline analysis has examined these new strategies as complementary and synergistic, or even future alternative to traditional antibacterial agents (i.e., small molecules) [13].

Among the 78 products identified in the clinical pipeline, 34 are nontraditional agents in a different stage of development: two are in the Marketing, Authorization, Application/New Drug Application (MAA/NDA) stage, five products in Phase 3, eleven in Phase 2, and five in Phase 1/2.

Nontraditional agents encompass different categories from phage-based therapies to monoclonal antibodies, antimicrobial peptides and antibacterial enhancers. Due to the wide range of approaches pursued in the nontraditional pipeline, these agents hold a great potential to curb AMR.

Although very distinct from each other in their nature, for the most part, traditional and nontraditional agents should be considered comparable when it comes to the need to adhere to predictable regulatory requirements for their development and potentially their approvals [14].

During the WHO Technical Advisory Group meeting on antibacterial research and development held on 29 and 30 November 2021, the scope of the nontraditional section was discussed, and it was decided that bacteriophage treatments will be included in the WHO pipeline analysis only if they were associated to a randomized controlled clinical trial while expanded access/compassionate use bacteriophage programs won’t be listed in the pipeline analysis. In addition, agents against biothreat pathogens will likely be considered in the 2023 update of the WHO antibacterial pipeline analysis and will also be evaluated in the context of the next update of the WHO Bacterial Priority Pathogen List (BPPL).

In conclusion, in the present scenario where traditional products have a limited lifespan before resistance emerges, non-conventional approaches offer opportunities to preserve new and old traditional agents, and to tackle AMR from different angles.

## Preclinical, traveling the valley of death

In the preclinical phase, there is a high turnover with almost one out of three developers lost every year. Half of the products in preclinical development target a single species, with 56 % targeting *Staphylococcus aureus, Pseudomonas aeruginosa and Mycobacterium tuberculosis.* This trend, which is also observed in the nontraditional space, requires availability of diagnostics at the point of care for optimal use, often unavailable outside of specialized healthcare facilities and poses a challenge in low-resource settings.

Overall, our analyses of the preclinical pipeline indicate a volatile landscape, with longer timeframes and challenging milestones to meet before potentially hitting the market. Thus, public, and private incentives, including new funding models becomes crucial in this space to continuously feed the clinical development with promising, safe, effective, on target and innovative new compounds [5,12].

## Final thoughts

The findings from 2021 WHO antibacterial pipeline analysis confirm that R&D is driven by private pharmaceutical companies, but the progress made since last year are extremely modest with only one product obtaining marketing authorization in China, one new product entering Phase 3 and five products entering Phase 1 and 7 products terminating their development. Smaller biotechs are facing major challenges, and the number of overall programs are still largely insufficient to properly address the burden of AMR.

Antibacterial agents in clinical development do not conclusively address the problem of extensive or pan-drug-resistant Gram-negative bacteria. Novel antibiotics targeting the critical WHO priority pathogens, are still lacking, in particular, carbapenem-resistant *A. baumannii* and *P. aeruginosa*. The pipeline also lacks oral antibiotic treatment options for extended spectrum β-lactamases (ESBLs) and CRE, which could allow for treatments outside the hospital-setting, and shorter treatment duration. In addition, optimized pediatric formulations of existing antibiotics are also needed, and there are barely any active pediatric development programs.

Overall, the recently approved antibacterial agents combined with the late-stage traditional agents under development are insufficient to address the enormous threat posed by AMR. More novel compounds that address unmet clinical needs are needed. These compounds must elude known mechanisms of drug resistance, and fit into modern reimbursement mechanisms.

Global public health efforts continue to focus on the relentless COVID-19 pandemic; resources are further diverted from other public health priorities, including AMR. This has also impacted clinical and preclinical developments, which have been substantially halted by the COVID-19 outbreak, with most programmes incurring considerable costs and delays likely for years to come.

A global response is needed to regain the time lost in the race against the silent AMR pandemic lurking in the shadows, waiting for its turn.
